# The genetic structure and demographic history of *Zabelia tyaihyonii*, endemic to Korean limestone karst forests, based on genome‐wide SNP markers

**DOI:** 10.1002/ece3.10252

**Published:** 2023-07-03

**Authors:** In‐Su Choi, Eun‐Kyeong Han, Martin F. Wojciechowski, Tae‐Im Heo, Jong‐Soo Park, Jong‐Cheol Yang, Amarsanaa Gantsetseg, Kyeong‐Sik Cheon, Ichiro Tamaki, Jung‐Hyun Lee

**Affiliations:** ^1^ Department of Biological Sciences and Biotechnology Hannam University Daejeon Korea; ^2^ Department of Biology Education Chonnam National University Gwangju Korea; ^3^ School of Life Sciences Arizona State University Tempe Arizona USA; ^4^ Division of Baekdudaegan Biodiversity Conservation Baekdudaegan National Arboretum Bonghwa Korea; ^5^ Division of Botany Honam National Institute of Biological Resources Mokpo Korea; ^6^ Department of Biological Science Sangji University Wonju Korea; ^7^ Gifu Academy of Forest Science and Culture Gifu Japan

**Keywords:** conservation, demographic history, limestone karst forests, MIG‐seq, *Zabelia tyaihyonii*

## Abstract

Similar to the global phenomenon, many plant species endemic to Korean limestone karst forests are at risk of extinction due to human intervention. *Zabelia tyaihyonii* is a familiar shrub, called “Hardy abelia” and “Fragrant abelia” growing in the karst forests of Korea, where it is one of the most threatened species. We investigated the genetic structure and demographic history of *Z. tyaihyonii*, which allow us to develop appropriate conservation and management strategies. The genetic structure was evaluated using a total of 187 samples from 14 populations, covering the entire distribution of *Z. tyaihyonii* in South Korea. We utilized 254 and 1753 SNP loci obtained via MIG‐seq (Multiplexed ISSR Genotyping by sequencing) for structure and demographic analyses, respectively. The population demographic modeling was performed with site frequency spectrum. To gain further historical insights, we also employed ENM (Ecological Niche Modeling). We found two distinct clusters (CLI and CLII) of ancient origin (ca. 490 ka). Despite CLII experiencing a more severe bottleneck, both clusters showed similar levels of genetic diversity, indicating mutual historical gene flow. Their historical distribution range seems to have changed very little. We proposed a historical distribution scenario for *Z. tyaihyonii*, taking into account its intrinsic factors, and emphasized a more complex response to Quaternary climate change beyond simple allopatric speciation models. These findings provide valuable insights for conservation and management strategies for *Z. tyaihyonii*.

## Graphical abstract

*Zabelia tyaihyonii* is a familiar shrub, called “Hardy abelia” and “Fragrant abelia” growing in the karst forests of Korea, where it is one of the most threatened species. We explored the evolutionary history and demographic dynamics for populations of *Z. tyaihyonii* using genome‐wide SNP markers. Here, we provide effective strategies on how to optimally conserve the historical genetic determinants of *Z. tyaihyonii*, known to have adaptively evolved on calcareous soils.

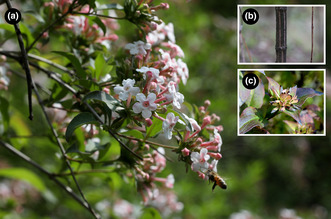

## 1 INTRODUCTION

The population genetic structure for a given plant species could be influenced by local differences in various extrinsic factors, such as availability of pollinators (Han et al., 2023), seed dispersers (Calviño‐Cancela et al., 2012), soil components (Nagasawa et al., 2020; Yamamoto et al., 2020), and climate conditions (Bisbing et al., 2021; Galliart et al., 2019). The degree of gene flow among populations is also considered a primary factor in shaping the population genetic structure (Tamaki et al., 2021). Although the relative importance of each factor may spatiotemporally change, the patterns of genetic differentiation observed in the majority of plants reflect historical, rather than current factors, particularly in East Asia (e.g., Cho et al., 2020; Han et al., 2020; Kikuchi & Osone, 2021). The population establishment history with demographic events is also associated with the Quaternary climatic oscillations that periodically affected both habitat fragmentation and connectivity, as well as geographical distribution range (Chung et al., 2017; Hewitt, 2000, 2003). The determinants of genetic variation obtained by this process may play a key role in the persistence of a species, by enabling the populations to adapt to rapidly changing environments (Hohenlohe et al., 2021; Médail & Baumel, 2018). Therefore, revealing the genetic structure of plant species and identifying the adaptive significance of specific populations can be the basis for establishing effective conservation strategies.

To minimize the extinction risks of populations, there are a growing number of population‐scale conservation studies that infer their evolutionary histories. With advances in next‐generation sequencing technology, various genetic frameworks have been applied to identify genetic information of a species underlying conservation managements (e.g., GBS, Lee et al., 2022; RAD‐seq, Stojanova et al., 2020). MIG‐seq (Multiplexed ISSR Genotyping by sequencing) is one of the various genotyping by sequencing (GBS) methods and can be an ideal technique for producing large datasets cost‐effectively at the population level (Suyama & Matsuki, 2015). It is advantageous when inferring past demographic events that might leave strong genetic footprints (Ishii et al., 2022; Yoichi et al., 2021).

**FIGURE 1:**
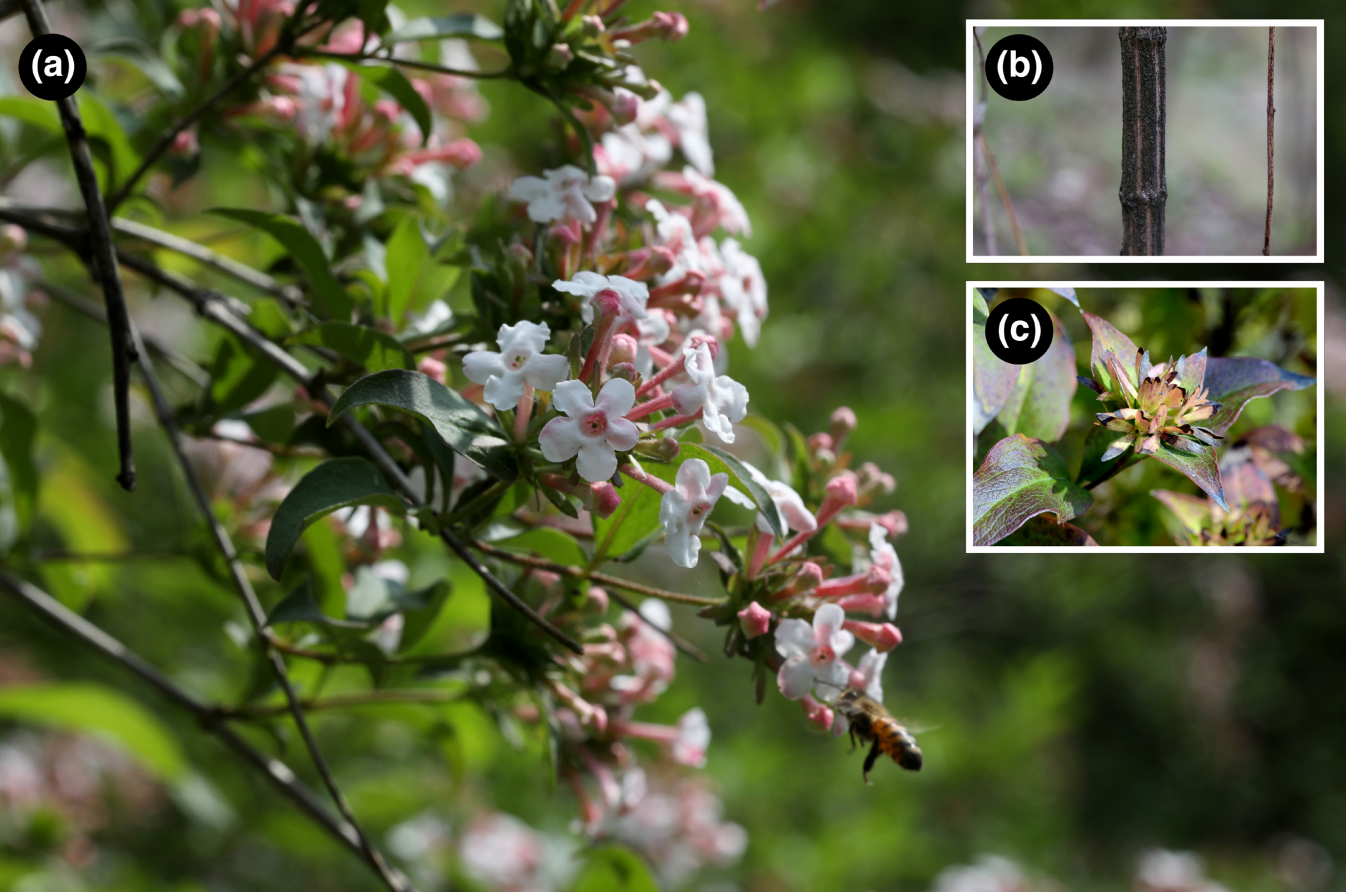
*Zabelia tyaihyonii* in its natural habitat. (a) flowering individual, (b) stem, (c) fruits (achene).

*Zabelia tyaihyonii* (Nakai) Hisauti & H. Hara is a temperate deciduous, broad‐leaved shrub in the family Caprifoliaceae that is endemic to the dry karst forests on the Korean Peninsula (Jeong et al., 2007; Figure 1). This species is known to have adaptively evolved on calcareous soils (Kim et al., 2021). *Z. tyaihyonii* has potential value for landscaping and gardening because of its colorful flowers (pale pink), excellent fragrance, and strong cold tolerance (Kim et al., 2022; syn. *Abelia mosanensis* T.H. Chung ex Nakai, see Rose, 2013), and has been traded under the names “Hardy abelia” and “Fragrant abelia”, since its introduction in the United States in 1989 (Nam et al., 2021; Sim & Seo, 1995). In South Korea, the Korea Forest Service has listed *Z. tyaihyonii* as an endangered species due to its extreme rarity and limited distribution in the karst forests (Korea National Arboretum, 2012). Nevertheless, concerns about their habitat destruction due to the mining industry are constantly emerging, as well as the over‐collecting of specimens for commercial purposes (Chae et al., 2023; Jeong et al., 2007; Kim et al., 2022).

The Korean limestone karsts are disjunctively separated on a smaller scale by non‐calcareous substrata, forming two zones, one each in South Korea and North Korea. Although *Z. tyaihyonii* is considered an endangered species in North Korea (Ju et al., 2016), little is known about its current status, and it is most likely not being protected in any way in North Korea. The karst forests in South Korea are home to many species, and about 100 vascular plant species grow preferentially in the limestone forests (Chung et al., 2013). In a given region, the accumulating genetic information for plant species can prove useful in setting conservation priorities (Chung et al., 2017, 2018). However, knowledge of the Korean limestone karsts remains inadequate for such purposes. Maintaining biodiversity for the karst forests can be challenging without conservation plans based on sufficient genetic data. A few population‐scale genetic studies have been conducted [*Goodyera rosulacea* Y.N. Lee: Chung & Chung, 2010; *Forsythia ovata* Nakai and *Forsythia saxatilis* (Nakai) Nakai: Chung et al., 2013; *Saussurea chabyoungsanica* Im: Jeong et al., 2012]. Also, previous studies of *Z. tyaihyonii* have sampled a very narrow range, and lacked historical inferences (Jeong et al., 2007; Kang et al., 2023). Therefore, our insight into its effective conservation is very limited.

A number of studies have been conducted to explore the effects of changes in geographical distribution due to Quaternary climatic fluctuations on the genetic diversity of temperate organisms, usually warm‐adapted (Petit et al., 2008). As a result, the focus of these studies has predominantly been on the latitudinal movements of warm‐adapted, species (e.g., Jin et al., 2021; Lee et al., 2013). The limited perspective has hindered our understanding of the diverse behavioral patterns exhibited by various species. To broaden our knowledge, it is essential to investigate the potential vertical movement and other factors of cold‐tolerant temperate plant species within specific areas (Cho et al., 2020). Therefore, it may be important to consider what historical distributional changes may have occurred for our target species that have evolved to adapt to limited limestone zones.

Ecological niche modeling (ENM) provides a powerful framework for investigating the interplay between ecological and evolutionary factors that have contributed to the genetic diversity of populations (Alvarado‐Serrano & Knowles, 2014). This approach is particularly useful in understanding the distribution and genetic structure of populations during Quaternary glacial cycles, even at small spatial scales (Cho et al., 2020; Jin et al., 2021). To shed light on the historical migration patterns of *Z. tyaihyonii*, we utilized ENM and population modeling techniques, including site frequency spectrum analysis. This will allow us to gain a better understanding of the factors that have shaped the genetic makeup of this species.

In this study, we aimed to explore the evolutionary history and demographic dynamics for populations of *Z. tyaihyonii* in South Korean karst forests. The specific goals of the present study are (1) to investigate their genetic diversity and structure, (2) to evaluate the evolutionary potential and conservation value to the populations from inferring the demographic history, and (3) to provide effective conservation guidelines for the recovery and management from a long‐term conservation genetics perspective.

## 2 MATERIALS AND METHODS

### 2.1 Study species and population sampling

Lasting problems have arisen with the use of the scientific name for this distinct and apparent taxon, *Z. tyaihyonii*. Controversy exists over whether the taxa distributed in the Korean Peninsula should be treated as the genus *Zabelia* (Rehder) Makino or *Abelia* R. Br. (Chung & Sun, 1984; Paik & Lee, 1989). However, a recent phylogenetic study suggested that *Zabelia* is a monophyletic group not closely related to *Abelia* (Jacobs et al., 2010). This is one of the reasons we recognize target species as *Z. tyaihyonii*. Nakai first reported *Abelia tyaihyonii* (= *Z. tyaihyonii*) collected in South Korea and *Abelia mosanensis* [= *Zabelia mosanensis* (T. H. Chung ex Nakai) Hisauti & H. Hara] collected in North Korea (Nakai, 1921, 1926, respectively). *Zabelia mosanensis* was described as distinct from *Z. tyaihyonii* by the features of having larger flowers (corolla and calyx) with hairs on the filaments. However, distinguishing these two taxa is inherently difficult because the reference specimens of *Z. tyaihyonii* were collected during the fruiting season (Sun, 1999). Moreover, *Z. tyaihyonii* also have hairs on their filaments (Nam et al., 2021). There is no evidence that *Z. mosanensis* has been found anywhere other than in *Z. tyaihyonii*'s habitats. Consequently, *Z. mosanensis* is regarded as a synonym of *Z. tyaihyonii* (Sun, 1999), that view is well maintained among Korean researchers (Chae et al., 2023; Kang et al., 2023; Won & Kim, 2007).

We collected 187 leaf samples of *Z. tyaihyonii* from the 14 populations in the limestone region, representing its entire distribution of South Korea. Only one leaf sample was collected per individual to reduce negative impacts on the species. Collected leaf samples were dried in silica gel on site and stored at the Biology Education lab, Chonnam National University. Total genomic DNA was extracted from the samples using the DNeasy Plant mini kit (Qiagen) according to the manufacturer's instructions. The concentration of extracted DNA was determined using Nano‐300 micro spectrophotometer (Allsheng), and diluted to 15 ng/μL to obtain the same concentration of template DNA in each sample.

### 2.2 MIG‐seq analysis

To obtain genome‐wide single‐nucleotide polymorphism (SNP) data, we used the MIG‐seq method. Preparation of the MIG‐seq library was conducted under standard conditions, according to Suyama et al. (2022), without a dark cycle. The first PCR was performed using the MIG‐seq primer set‐1 to amplify ISSR regions from genomic DNA. The first PCR products were identified by visualization on 0.8% agarose gels and then used as templates for the second PCR (tailed PCR). The second PCR was conducted to add individual indices to each sample using the indexed forward and reverse primers. Each second PCR product was pooled as a single mixture library, and the mixture was purified with a QIAquick PCR purification Kit (Qiagen). The purified mixture was size‐selected in the range of 350–650 bp using the BluePippin system (Sage Science). We sequenced a dual‐indexed library containing all samples using Illumina HiSeq X at Macrogen, and 151 bp paired‐end reads were obtained. The MIG‐seq raw reads were grouped into those from individual samples using the index read option of the sequencer.

### 2.3 SNP detection

For quality control of the raw reads, the first 17 bp, including partial primer sequences at the 5′ end of each raw read, were trimmed (Nishimura et al., 2022) using the program fastx_trimmer in the FASTX‐Toolkit 0.0.14 (http://hannonlab.cshl.edu/fastx_toolkit/) so that the length of each read became 134 bp. The low‐quality reads were removed using the program quality_filter in the FASTX‐Toolkit, and extremely short sequence reads were then removed by TagDust 1.12 (Lassmann et al., 2009). The quality‐filtered reads were analyzed with Stacks v.2.5 (Catchen et al., 2013) to detect SNPs. The stacks in each sample were built by de novo assembly with the option of minimum depth coverage required to create a stack (m) = 3 and maximum difference in nucleotides allowed between stacks (M) = 2. The SNP dataset was created using the ‘populations’ command. The minimum number of populations at a locus (‐p) was set to 14, the total number of populations. We also included only the first SNP per locus (‐‐write‐single‐snp) to avoid bias towards specific stacks, and set the minimum minor allele frequency to 0.03 (‐‐min‐maf 0.03) and maximum observed heterozygosity to 0.60 (‐‐max‐obs‐het 0.60). The polymorphic loci values for the Stacks were determined under percent samples limit per population (‐r) = 1.00, 0.90, 0.85, and 0.80, respectively. The optimal values of SNP dataset were determined when the number of polymorphic loci appeared to be maximized or stabilized around the maximum.

### 2.4 Analysis of population genetics statistics and population structure

Population genetics statistics were estimated based on MIG‐seq SNP data using GenAlEx version 6.502 (Smouse & Peakall, 2012). These included the number of private alleles (*P*_A_), observed heterozygosity (*H*_O_), expected heterozygosity (*H*_E_), and inbreeding coefficient (*F*_IS_). Nucleotide diversity (*π*) was calculated using the ‘populations’ command of Stacks. Allele richness (*A*_R_) and genetic differentiation among populations were determined according to the method of Weir and Cockerham (1984), using FSTAT version 2.9.4 (Goudet, 1995).

To infer the population structure of *Z. tyaihyonii*, we used a Bayesian clustering approach implemented in STRUCTURE version 2.3.4 (Pritchard et al., 2000) using 100,000 Markov Chain Monte Carlo (MCMC) iterations (10,000 burn‐in, with admixture). The simulation used 20 iterations, with *K* = 1–14 clusters. We determined the optimal number of clusters, *K* value, using STRUCTURE HARVESTER (Earl & VonHoldt, 2012). CLUMPP version 1.1.2 (Jakobsson & Rosenberg, 2007) with the Greedy algorithm was used to combine the membership coefficient matrices (Q‐matrices) from 1000 iterations using random input orders. We also conducted a principal coordinate analysis (PCoA) to understand the genetic relationships among individuals and among populations using the covariance standardized approach of pairwise Nei's genetic distances in GenAlEx. To visualize relationships among populations, we constructed a split network using SplitsTree4 version 4.14.5 with the Neighbor‐Net algorithm (Huson & Bryant, 2006). We performed Mantel test (Mantel, 1967) to test for the presence of isolation‐by‐distance (IBD) using GenAlEx. We ran the analysis with 999 random permutations to evaluate the relative correlation of genetic differentiation and geographical distance between populations.

### 2.5 Inference of population demography

**FIGURE 2:**
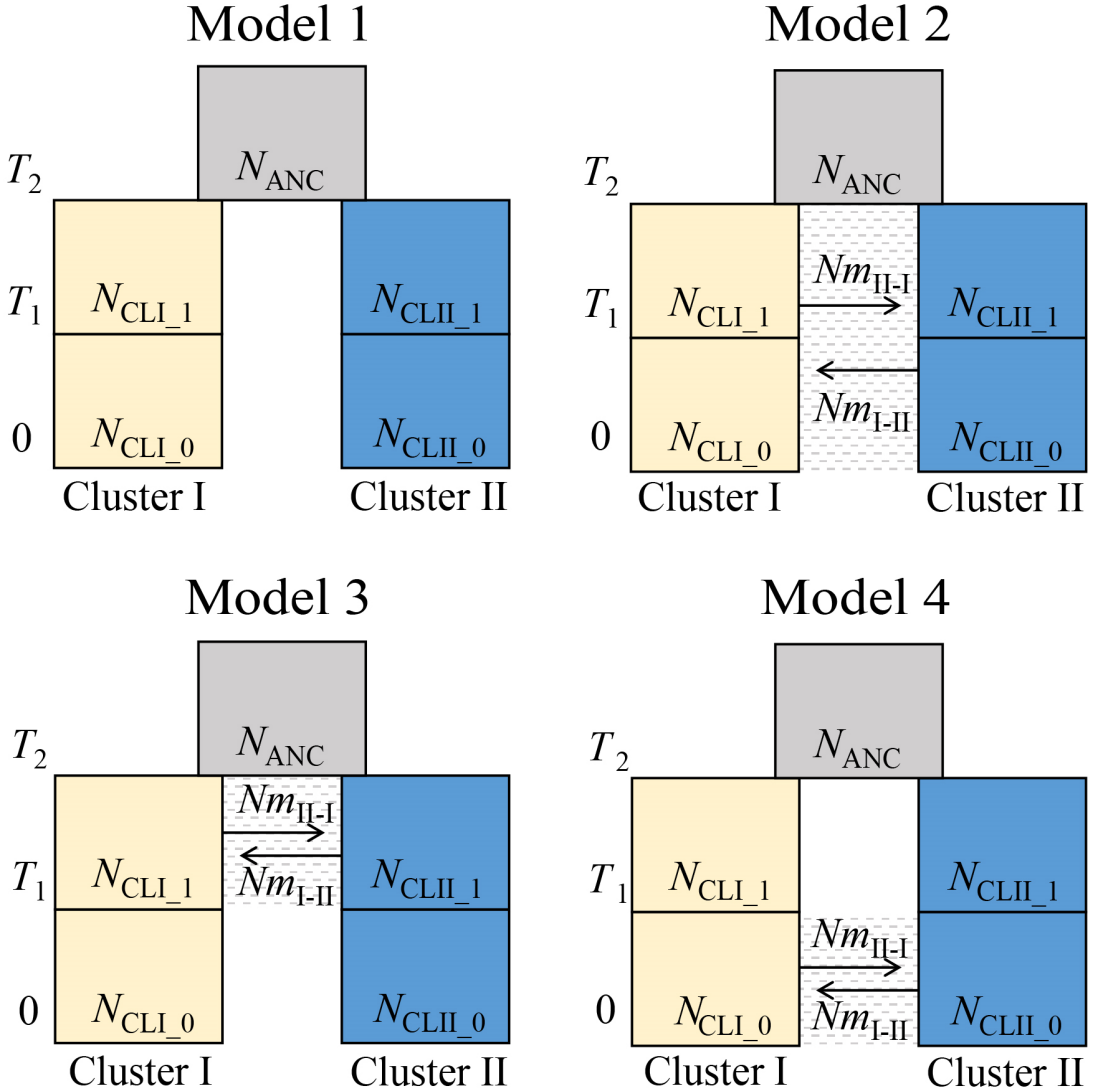
Comparison of four two‐population divergence models. *N*, effective population size; *Nm*_i‐j_, number of migrants per generation from cluster *j* to *i* (its direction is forward‐in‐time); *T*, event time. The period shown in dashed lines assumes migration between clusters.

In order to identify divergence times, patterns of migrations, and population size changes, a coalescent based maximum likelihood analysis estimating parameters of the population demographic model was performed with fastsimcoal2 version 2709 (Excoffier et al., 2021). We assumed two populations, cluster I and cluster II, based on the results of our Structure analysis. Four two‐population divergence models were constructed (Figure 2). Model 1 (no‐migration model) assumed no‐migration between populations. Models 2 (isolation with migration model) assumed continuous migration during from current to *T*_2_. Model 3 (ancient migration model) assumed ancient migration during from *T*_1_ to *T*_2_. Model 4 (secondary contact model) assumed recent migration during from current to *T*_1_. We also assumed different population size change in different three time periods, from current to *T*_1_, from *T*_1_ to *T*_2_ and before *T*_2_.

To make the input data for fastisimcoal2, as we need information that how many SNPs were obtained from sequences of certain lengths, SNPs were re‐extracted using populations of stacks with options of ‐p 2 ‐r 0.80 ‐‐max‐obs‐het 0.60 ‐‐min‐maf 0.0. The re‐extracted data was constructed from 242,107 all sites, 4178 variant sites (SNPs) and 1753 loci (stacks). Two dimensional minor allele site frequency spectrum (2D‐mSFS) was calculated from the .vcf file with our own R script (https://github.com/garageit46/2D‐msfs‐R). Missing data was compensated by a bootstrapping within the cluster.

The likelihood of each model was maximized from 50 random starting values, 40 expectation‐conditional‐maximization (ECM) optimization cycles, and 100,000 coalescent simulations. Mutation rate was set to 5.39 × 10^−8^ per site per generation, which was estimated in a woody shrub, *Lonicera oblata* (7.7 × 10^−8^ per site per year × 7 years per generation; Mu et al., 2022). We calculated Akaike's information criterion (AIC) and selected the model with the lowest AIC value as the best model. Goodness of the fitness of the best model was checked by comparison of observed and simulated 2D‐mSFS visually. Confidence interval of the best model was calculated by a parametric bootstrapping. We simulated the model by fastsimcoal2 with the maximum likelihood estimate parameter values 100 times and obtained its 2D‐mSFS. Using the simulated 2D‐mSFS as an input data, parameters of the best model were re‐calculated with the observed parameter values as a stating value, 15 ECM cycles and 100,000 coalescent simulations. Finally, 95% confidence intervals (CI) were calculated from the obtained parameter values. We assumed a generation time of 20 years for *Z. tyaihyonii* based on the fact that its phylogenetically closely related and commonly cultivated taxon, *Abelia* × *grandiflora* (Andrè) Rehd., takes 10–20 years to reach its ultimate height (Talhouk et al., 2015). Considering that the growth of *Z. tyaihyonii* in nature may be considerably slower than in garden conditions, we have maximized the generation time to 20 years. Consequently, twenty years per generation was used to convert an event time from generations ago to years ago.

### 2.6 Ecological niche modeling

We performed ecological niche modeling analysis with climatic and lithological data to estimate the past distribution of *Z*. *tyaihyonii*. We first developed a model of current distribution based on occurrence data, and then past distribution was estimated by projecting the model to paleo‐climatic data. We used only 14 occurrence data obtained from our investigations in the analysis because of the rareness of the species. We downloaded 19 bioclimatic variables from Climatologies at High resolution for the Earth's Land Surface Areas (CHELSA, http://chelsa‐climate.org; Karger et al., 2017) for two periods (the present, Last Glacial Maximum; LGM). Three bioclimatic variable sets of LGM are based on three General Circulation Models (GCMs): the Community Climate System Model version 4 (CCSM4; Gent et al., 2011), the Earth System Model based on the Model for Interdisciplinary Research On Climate (MIROC‐ESM; Watanabe et al., 2011), and the Max Planck Institute for Meteorology Earth System Model (MPI‐ESM‐P). We also use lithological data of GLiM (Hartmann & Moosdorf, 2012) to reflect critical ecological conditions of the species into the distribution model. To avoid multicollinearity, the bioclimatic variables showing high Spearman correlation efficient (>0.8) by using SDMtoolbox 2.4 (Brown et al., 2017) were excepted. After preliminary modeling analysis using the remaining bioclimatic variables, the one revealing a flat response curve was also excluded. Therefore, four bioclimatic variables and one lithological data were used in developing distribution models targeting 33–43°N latitude and 123–132°E longitude. To reduce the uncertainty resulting from using the different GCMs, the LGM distribution models corresponding to three GCMs were averaged (Wiens et al., 2009). We use Maxent 3.4.4 (Merow et al., 2013) with batch mode, and it was set with cross‐validation, jackknife tests, regularization parameter 2, 20 replicates, cloglog output, random seeds, and 10,000 background points and 2000 iterations.

## 3 RESULTS

### 3.1 Genetic diversity and genetic structure

In total, we obtained 462,669,080 raw reads from the 187 samples of *Z. tyaihyonii*. The average number of raw reads per sample was 2,474,166 (ranging from 934,936 to 4,403,402). After the quality control of the raw reads, 234,710,807 reads were retained for SNPs calling. Sample coverage for genotyping of *Z. tyaihyonii* was 32.36x. From the population program in Stacks with the parameter combination of ‐p 14 ‐‐max‐obs‐het 0.60 ‐‐min‐maf 0.03 and write‐single‐snp, a total of 3, 21, 47, and 254 SNPs (loci) were detected under r = 1.00, 0.90, 0.85, and 0.80, respectively. Genetic diversity was estimated from 254 SNPs.

Genetic diversity parameters, evaluated at the population and pooled cluster levels for all 187 individuals of *Z. tyaihyonii*, are shown in Table 1. Genetic diversity of *Z. tyaihyonii* populations showed relatively similar allelic richness, expected heterozygosity, and nucleotide diversity (*A*_R,_ *H*_E_, and π) across all populations except for KY_H1 and CJ_S6. At the population level, *A*_R_ and *H*_E_ ranged from 1.217 to 1.570 (mean 1.471) and 0.086 to 0.198 (mean 0.167), respectively. π ranged from 0.093 to 0.202 (mean 0.175). At the pooled cluster level, the levels of genetic diversity were almost equivalent between cluster I (*A*_R_ 1.923, *H*_E_ 0.202, *π* 0.231) and cluster II (*A*_R_ 1.888, *H*_E_ 0.204, *π* 0.239). Also, each of the two clusters harbored private alleles (28 and 17, respectively).

**TABLE 1:** Genetic diversity parameters of the 14 populations of *Zabelia tyaihyonii* in Korea based on 254 SNPs.

ID	Location	*N*	*N*_A_	*P*_A_	*A*_R_	*H*_O_ (SE)	*H*_E_ (SE)	*π*	*F*_IS_
Cluster I									
KY_H1	Sincheon‐ri, Yeongwol‐gun, Gangwon‐do	6	1.220	0	1.217	0.193 (0.024)	0.101 (0.012)	0.110	−0.854
KY_B2	Bukssang‐ri, Yeongwol‐gun, Gangwon‐do	16	1.638	0	1.505	0.177 (0.012)	0.184 (0.012)	0.190	0.020
KY_B4	Bukssang‐ri, Yeongwol‐gun, Gangwon‐do	17	1.630	0	1.487	0.193 (0.015)	0.179 (0.012)	0.185	−0.037
KY_B5	Bukssang‐ri, Yeongwol‐gun, Gangwon‐do	9	1.626	0	1.540	0.185 (0.013)	0.182 (0.011)	0.193	−0.020
KY_B6	Bukssang‐ri, Yeongwol‐gun, Gangwon‐do	14	1.646	0	1.514	0.188 (0.013)	0.178 (0.011)	0.185	−0.049
KY_C2	Changwon‐ri, Yeongwol‐gun, Gangwon‐do	22	1.591	1	1.453	0.168 (0.013)	0.165 (0.011)	0.169	−0.023
KY_C5	Changwon‐ri, Yeongwol‐gun, Gangwon‐do	15	1.665	0	1.507	0.178 (0.013)	0.179 (0.011)	0.186	0.013
CJ_S6	Sinhyeon‐ri, Jecheon‐si, Chungcheongbuk‐do	7	1.244	0	1.231	0.114 (0.015)	0.086 (0.010)	0.093	−0.227
Population mean	—	1.532	0.166	1.432	0.175	0.157	0.164	−0.074	
Pooled cluster	106	1.933	28	1.923	0.176	0.202	0.231	0.114	
Cluster II									
CD_M1	Sangsi‐ri, Danyang‐gun, Chungcheongbuk‐do	7	1.555	0	1.513	0.179 (0.014)	0.173 (0.011)	0.187	−0.049
CD_M3	Sangsi‐ri, Danyang‐gun, Chungcheongbuk‐do	8	1.606	0	1.544	0.186 (0.013)	0.183 (0.011)	0.195	−0.023
CD_M4	Sangsi‐ri, Danyang‐gun, Chungcheongbuk‐do	23	1.772	0	1.570	0.195 (0.011)	0.198 (0.010)	0.202	0.011
CD_M5	Sangsi‐ri, Danyang‐gun, Chungcheongbuk‐do	10	1.650	0	1.553	0.181 (0.012)	0.190 (0.011)	0.200	0.027
CD_M6	Hasi‐ri, Danyang‐gun, Chungcheongbuk‐do	7	1.500	0	1.464	0.184 (0.015)	0.165 (0.012)	0.178	−0.116
CD_M7	Dogok‐ri, Danyang‐gun, Chungcheongbuk‐do	26	1.642	0	1.491	0.187 (0.013)	0.178 (0.011)	0.181	−0.043
Population mean	—	1.621	0	1.551	0.185	0.181	0.191	−0.116	
Pooled cluster	81	1.890	17	1.888	0.179	0.204	0.239	0.094	

Abbreviations: *A*_R_, allelic richness; *F*_IS_, inbreeding coefficient; *H*_E_, expected heterozygosity; *H*_O_, observed heterozygosity; *N*, number of individuals; *N*_A_, number of alleles; *P*_A_, number of private alleles; SE, standard error; *π*, nucleotide diversity.

**FIGURE 3:**
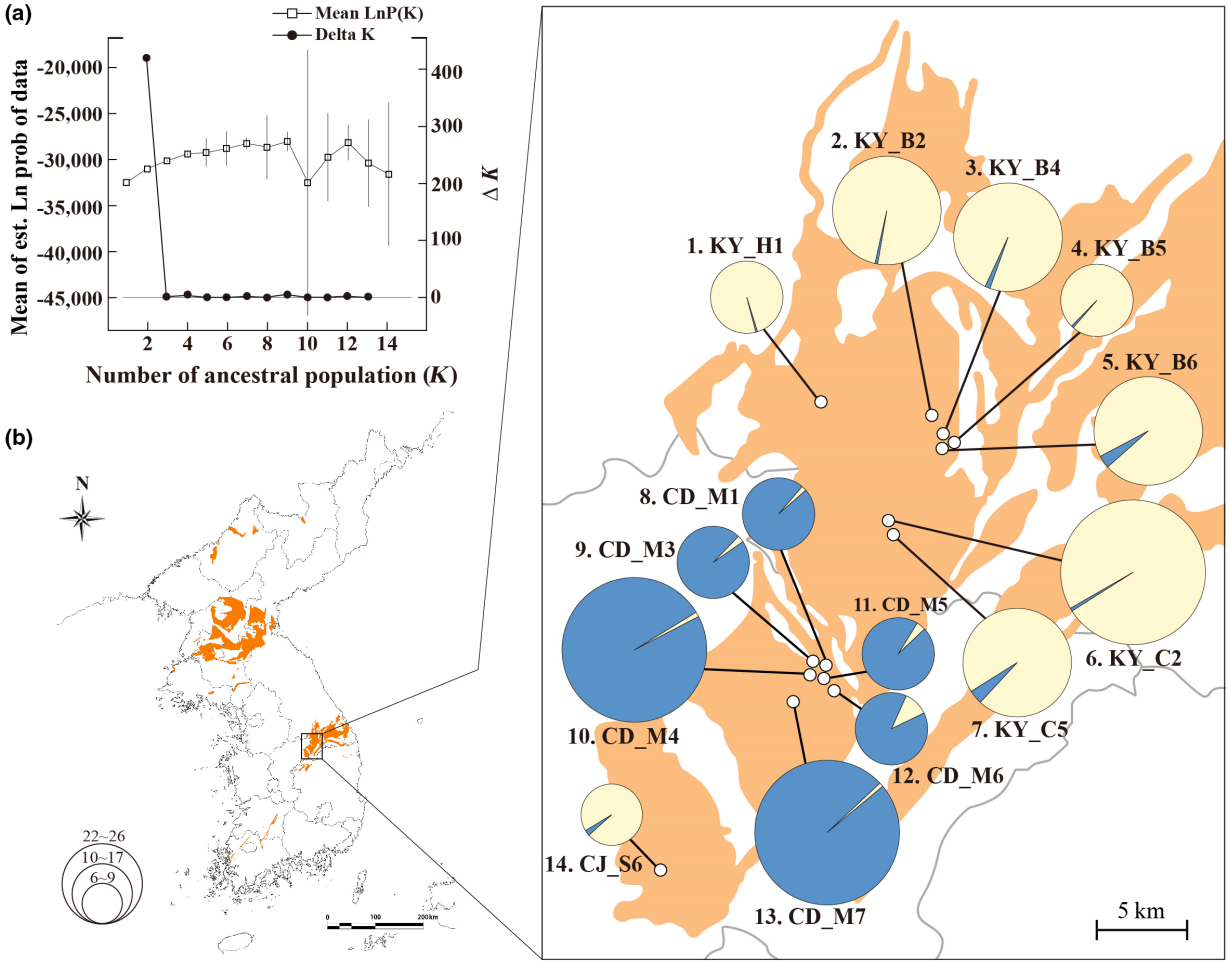
Genetic composition of *Zabelia tyaihyonii* populations using 254 SNPs. (a) Plots showing the rate of change in log‐likelihood probability and Δ*K* based on the estimated number of genetic clusters (*K*). (b) Genetic composition based on STRUCTURE clustering results (*K* = 2). Orange‐colored regions represent limestone areas.

**FIGURE 4:**
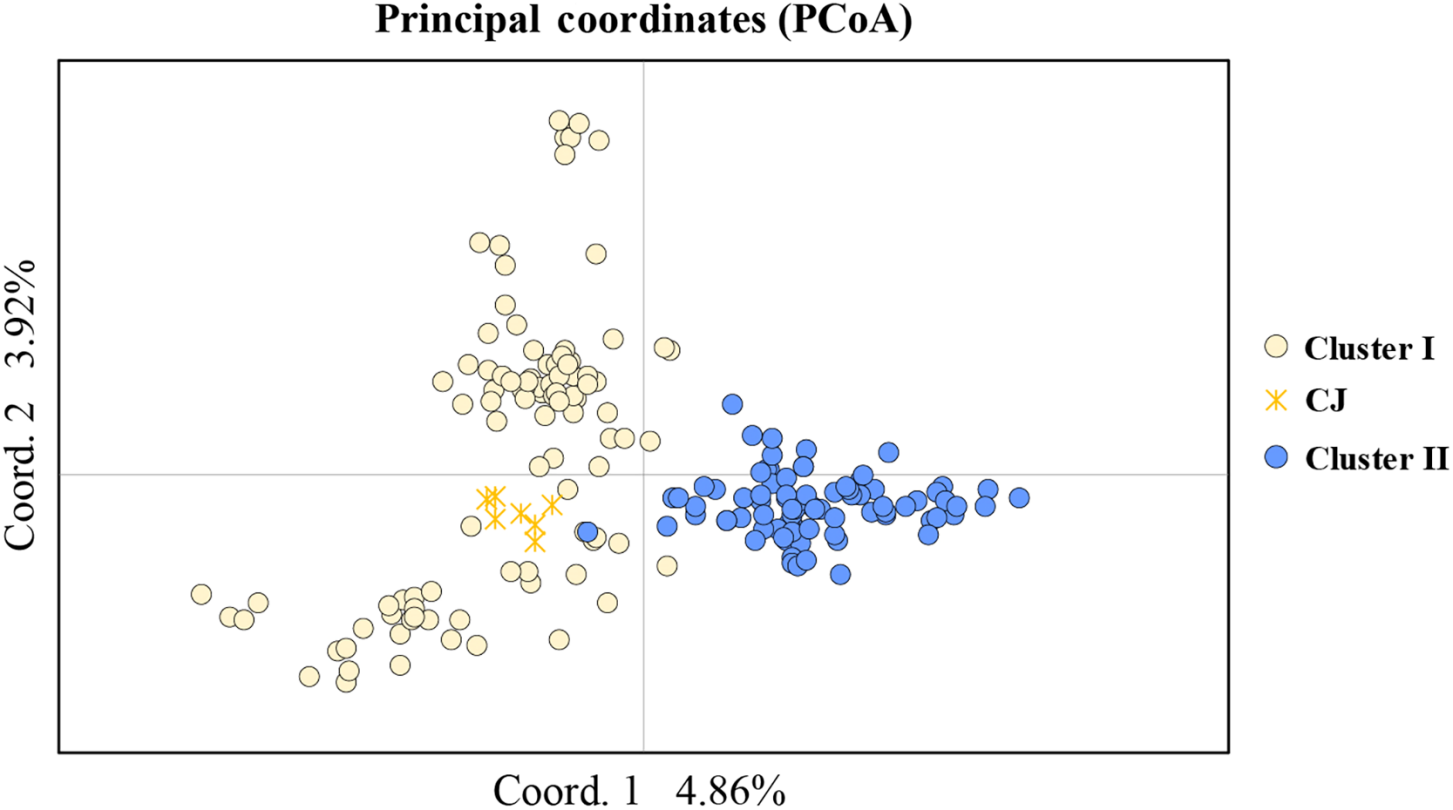
The scatter plot of principal coordinate analysis (PCoA) for *Zabelia tyaihyonii* individuals based on the 254 SNPs. Individuals are color‐coded by cluster. Individuals in the CJ population are highlighted with yellow asterisks.

**FIGURE 5:**
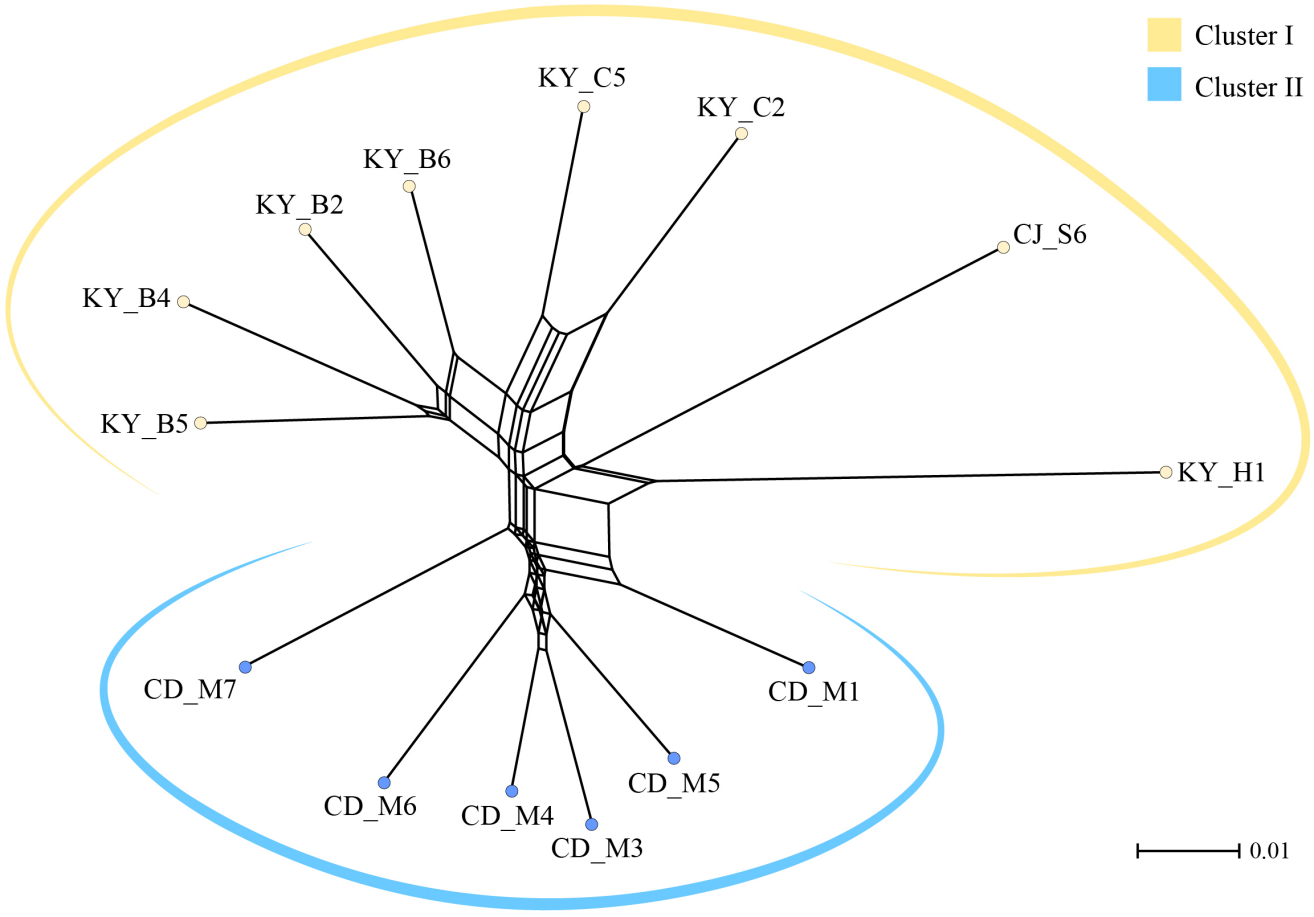
Phylogenetic networks constructed by the Neighbor‐Net method based on *Zabelia tyaihyonii* populations using 254 SNPs. Branch tips represent populations, and colors represent the two clusters according to genetic lineage. The scale bar indicates genetic distance.

To infer the population structure of *Z. tyaihyonii*, we performed STRUCTURE, PCoA, and Neighbor‐Net phylogeny analysis with 254 SNPs from MIG‐seq. The results clearly demonstrated that the two clusters are genetically distinct. The genetic structure strongly supported *K* = 2 as the optimal number of clusters for the datasets, based on Δ*K* statistics in the STRUCTURE analysis (Figure 3). At *K* = 2, the populations of *Z. tyaihyonii* were divided into two clusters, cluster I (CLI; KY‧CJ populations) and cluster II (CLII; CD populations). The results of the PCoA for individuals were not clearly separated, but were divided into two clusters based on similarity indices (Figure 4). The first two principal coordinates (coord. 1 and coord. 2) accounted for 4.86% and 3.92% of the variation, respectively, explaining 8.78% of the total variation. Neighbor‐Net phylogenetic network suggested that the two clusters are genetically well differentiated (Figure 5). Additionally, the isolation by distance (IBD) analysis carried out through the Mantel's correlation test demonstrated that *Z. tyaihyonii* exhibited a significant positive correlation (*R* ^2^ = .330, *p* = .003) between genetic differentiation and geographic distances (Figure S1).

### 3.2 Inference of population demography

Isolation with the migration model (model 2) was selected as the best model (Table 2). Goodness of fitness was visually confirmed by plotting observed and simulated 2D‐mSFSs (Figure S2). As the CIs were not duplicated, current effective population size of CLI (*N*_CLI_0_ = 5008, 95% CI: 3266–6873) was significantly larger than that of CLII (*N*_CLII_0_ = 1766, 95% CI: 1185–2673; Table 3). Both clusters showed significant recent reduction in population size at *T*_1_ (2.6 ka, 95% CI: 1.3–5.1 ka) because *N*_CLI_0_/*N*_CLI_1_ and *N*_CLII_0_/*N*_CLII_1_ were significantly smaller than 1.0. CLI also showed significant increase in population size at its divergence. Divergence time between the two clusters (*T*_2_) was 490 ka (95% CI: 292–671 ka). Strengths of migrations between clusters denoted by the number of migrants per generations (*Nm*) were significantly larger than 1.0 in both direction, and there was no statistically significant difference between directions, as their 95% CIs overlapped.

**TABLE 2:** Number of parameters (NP), log‐likelihood (LL; in log_10_ scale), Akaike's information criterion (AIC) and maximum likelihood estimates of parameters for two‐population divergence models.

Parameter	Model 1	Model 2	Model 3	Model 4
NP	7	**9**	9	9
LL	−21,333	**−21,240**	−21,246	−21,243
AIC	98,256	**97,832**	97,860	97,846
*N*_CLI_0_	6030	**5008**	1982	6032
*N*_CLII_0_	3607	**1766**	1780	3187
*N*_CLI_1_	16,757	**9429**	10,000	15,412
*N*_CLII_1_	4697	**7598**	5299	100,379
*N*_ANC_	12,835	**4480**	3907	9658
*T*_1_ (ka)	6.4	**2.6**	0.4	18
*T*_2_ (ka)	8.8	**490**	532	89
*Nm*_I–II_	—	**1.777**	2.235	2.528
*Nm*_II–I_	—	**2.141**	2.387	1.901

*Note*: The best model was shown in bold.

Abbreviations: *N*, effective population size; *Nm*_i–j_, number of migrants per generation from cluster j to i (its direction is forward‐in‐time); *T*, event time.

**TABLE 3:** Maximum likelihood estimate (MLE) and 95% confidence interval (CI) of parameters in the best two‐population divergence model (Model 2 in Figure 2).

Parameter	MLE (95% CI)
*N*_CLI_0_	5008 (3266–6873)
*N*_CLII_0_	1766 (1185–2673)
*N*_CLI_1_	9429 (7794–11,432)
*N*_CLII_1_	7598 (5581–10,148)
*N*_ANC_	4480 (1602–8036)
*T*_1_ (ka)	2.6 (1.3–5.1)
*T*_2_ (ka)	490 (292–671)
*Nm*_I‐II_	1.777 (1.437–2.202)
*Nm*_II‐I_	2.141 (1.744–2.353)
*N*_CLI_0_/*N*_CLI_1_ *	0.531 (0.316–0.889)
*N*_CLII_0_/*N*_CLII_1_ *	0.232 (0.140–0.383)
*N*_CLI_1_/*N*_ANC_ *	2.105 (1.214–6.032)
*N*_CLII_1_/*N*_ANC_	1.696 (0.755–4.635)

Abbreviations: CLI and CLII indicate cluster I and cluster II, respectively; *N*, effective population size; *N*_CLX_0_ and *N*_CLX_1_ indicate effective population size of cluster X during from current to *T*_1_ and from *T*_1_ to *T*_2_, respectively; *Nm*_i–j_, number of migrants per generation from cluster j to i (direction of migration is forward‐in‐time); *T*, event time.

* Significantly different from 1.0.

### 3.3 Ecological niche modeling

**FIGURE 6:**
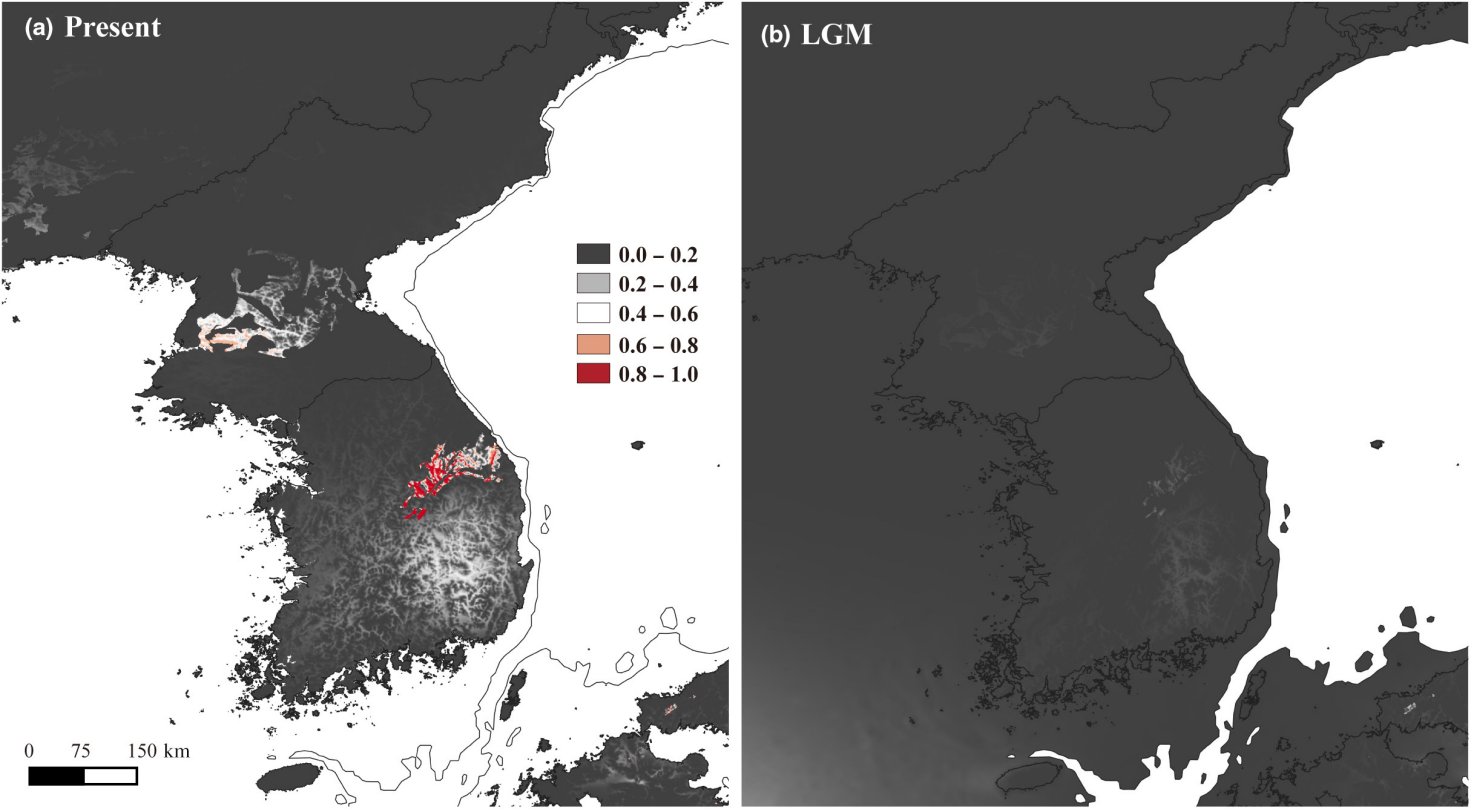
Predicted distributions of *Zabelia tyaihyonii* using bioclimatic and lithological variables during (a) the present and (b) the Last Glacial Maximum, LGM. Distributions predicted by ecological niche modeling; The distribution during the LGM was averaged from results using three General Circulation Models. The distribution during the LGM shows almost none suitable area in Korean Peninsula for *Z. tyaihyonii*.

Current predicted distribution of *Z*. *tyaihyonii* was made by using Maxent modeling and revealed high AUC (0.977). The variable showing the highest percent contribution is glim (lithological data; 52.4%) and second one is bio04 (temperature seasonality; 18.2%). The current predicted distribution shows that it is significantly associated with the limestone area in Korean Peninsula (Figure 6). The LGM distribution, which was projected and averaged from three GSMs, reveals that there was only a low suitable area (<0.6) for the species, which is also related to the limestone area.

## 4 DISCUSSION

We used the MIG‐seq technique to study the genetic diversity of *Z. tyaihyonii*, a karst‐restricted plant species in South Korea that is under threat. This allowed us to conduct a detailed genetic assessment of this endangered shrub in a small area. Our analysis showed that there are two genetically distinct clusters, CLI and CLII of ancient origin (ca. 490 ka), and we were able to identify the historical and intrinsic factors that shaped their genetic composition. Despite CLII having a more severe bottleneck, both the clusters had similar levels of genetic diversity. This can be attributed to bidirectional historical gene flow that contributed to the genetic diversity of both groups without compromising their genetic uniqueness. We propose a historical distribution scenario for *Z. tyaihyonii* that considers its intrinsic factors, and highlights a more complex scenario that takes into account its response to Quaternary climatic fluctuations, beyond simple allopatric speciation models.

### 4.1 Population establishment history and genetic structure

We found that the *Z. tyaihyonii* populations are structured in two genetically different clusters, as shown in the STRUCTURE, Neighbor‐Net phylogeny, and PCoA analyses with a significant IBD pattern. However, genetic variation in the CJ_S6 population appears to be inconsistent with the geographical proximity of the two clusters in the limestone zones. This population is isolated from other populations by a physical barrier of non‐calcareous substrata. Despite this, the CJ_S6 population does not show unique genetic characteristics and instead forms a clade with geographically more distant populations within CLI, indicating genetic homogeneity. Because a previous study conducted by Kang et al. (2023) had limited coverage of CLII populations, it is challenging to directly compare their findings with our current study. The study demonstrated significant genetic divergence for the CJ_S6 population within the CLI populations. Nevertheless, taking all our findings into account, we propose that the single non‐conforming CJ_S6 population might have been established by a chance migration event in the relatively recently, possibly after the time of complete divergence between CLI and CLII.

We noted that ENM simulated in bedrock conditions revealed that the potential distribution converges to zero across the Korean Peninsula, as if *Z. tyaihyonii* populations were extinct during the LGM. This result may imply that their current distribution may have been largely unaffected by the Quaternary glacial–interglacial oscillations that contributed to genetic variation and geographic adequacy (Han et al., 2020; Tamaki et al., 2018, 2021). We suggest that the strong cold tolerance of *Z. tyaihyonii* made it possible to avoid forcing the southern retreat of the distribution range by climate cooling during glacial periods. Indeed, *Z. tyaihyonii* (= *Abelia mosanensis*) can withstand minimum temperatures from −28.9 to −23.3°C, thus it is considered cold hardy (as *Abelia mosanensis* in Rose, 2013). It is estimated that temperatures at the peak of the past glacial maximum were about 6 degrees cooler than today (Golledge et al., 2012; Hulton et al., 2002; Seltzer et al., 2021). Considering that the average of the last 28 years of the average minimum temperature of the coldest month in the limestone forests was −9.18°C (Yeongwol) and −10.96°C (Jecheon), respectively (Korea Meteorological Administration; https://data.kma.go.kr/), our idea regarding their persistent distribution is considered reasonable. It is, therefore, conceivable that the extant populations are descended from ancestor populations (at least 490,000 years ago) that had lived in the same (much not different) place as those of the present.

### 4.2 Genetic diversity and historical gene flow

We detected signs of a recent bottleneck in both CLI and CLII, which can be inferred from the change in the effective population size (Table 2). However, the degree of bottleneck was significantly stronger in CLII. A bottleneck event is almost universally accompanied by a loss of genetic diversity (e.g., Jacquemyn et al., 2010; Landergott et al., 2001; Zheng et al., 2012), and thus we might expect to observe lower genetic diversity in populations that have experienced a stronger bottleneck. Interestingly, our results show similar levels of genetic diversity between the two clusters at both the population and pooled sample levels. This suggests that there may be intrinsic or other external factors that have helped to maintain genetic diversity in CLII and potentially in this species as a whole.

The one of most plausible factors can be found in symmetric and persistent historical gene flow, as shown in our demographic analysis (Figure 5). Although the two genetic clusters are genetically distinct, there exists moderate level of historical gene flow. This level of gene flow is likely not sufficient to significantly impact the genetic distinctiveness of each cluster (e.g., Tamaki et al., 2018, 2021). Another possibility can be on their flexible reproductive system of *Z. tyaihyonii* (sexual and asexual reproduction). Due to their flowering time (May), strong floral scent, and floral structure, these shrubs are considered to be active in cross‐fertilization, but often give rise to generations through vegetative reproduction (Jeong et al., 2007). Indeed, several closely related species, including this species, can be readily cultivated by vegetative cuttings for horticultural (e.g., *Abelia* × *grandiflora*: Loconsole et al., 2022) and conservation purposes (e.g., *Z. tyaihyonii*: Yoon et al., 2022). Availability of asexual reproduction can mitigate the negative genetic impact of population bottlenecks (Roman & Darling, 2007). Despite severe bottlenecks, the predominant asexual populations often exhibit similar levels of genetic diversity in terms of heterozygosity and allele richness when compared to their counterparts [e.g., *Glycyrrhiza inflata* Batalin: Yang et al., 2016; *Lindera glauca* (Siebold & Zucc.) Blume: Zhu et al., 2020]. Consequently, we believe that accumulative historical gene flow and the flexible reproductive system of *Z. tyaihyonii* could mitigate the negative effects of locally occurring or varying strength bottlenecks. This would have allowed for the maintenance of similar genetic diversity throughout the species' distribution range.

### 4.3 Conservation implications

Based on full range of analyses, we recommend that conservation strategies for *Z. tyaihyonii* populations in South Korea should be developed to maintain the unique genetic identity of each cluster. Additionally, since it is uncertain whether other *Z. tyaihyonii* populations exist in North Korea, it is important to consider that the few remaining populations in South Korea may represent the entire distribution range of this species. This fact should be a fundamental consideration when contemplating industrial development in specific habitats of this species. The possibility of the species existing in North Korea could potentially make it more vulnerable to extinction of this shrub by providing a more favorable perspective towards development.

Random genetic drift tends to decrease evolutionary adaptation and to fix harmful alleles to populations, making them maladapted to a changing environment (Stetter et al., 2018; Weeks et al., 2016). Nevertheless, we do not believe that increasing genetic diversity in these populations should be the primary focus of conservation strategies. The current level of genetic diversity in *Z. tyaihyonii* may be a strategic outcome that has contributed to their long‐term survival, potentially due to their flexible outcrossing and vegetative reproductive systems. Moreover, our analysis suggests that the uniform genetic diversity across the entire populations may be a result of historical environmental changes, rather than recent human‐induced destruction (e.g., Tamaki et al., 2016). Therefore, we propose that the current level of genetic diversity in these two clusters may be well‐suited for their long‐term survival.

From a long‐term conservation perspective, we highlight that *Z. tyaihyonii* populations in South Korea should be conserved and managed in a way that allows for natural migrations between the two clusters. Rather than considering the clusters as completely separate units, a strategy that promotes further increases in population size should be adopted. To prevent further isolation, land development in areas that could serve as corridors or migration routes between the clusters should be avoided. When selecting donor populations for each cluster, it is crucial to focus on genetic suitability rather than geographic proximity, especially for the CJ_S6 population.

## AUTHOR CONTRIBUTIONS

**In‐Su Choi:** Conceptualization (equal); data curation (lead); methodology (equal); writing – original draft (lead); writing – review and editing (equal). **Eun‐Kyeong Han:** Formal analysis (equal); methodology (equal); software (equal); visualization (lead). **Martin Wojciechowski:** Writing – review and editing (equal). **Tae‐Im Heo:** Conceptualization (equal); investigation (equal); project administration (equal); resources (equal). **Jong‐Soo Park:** Methodology (equal); software (equal); visualization (supporting). **Jong‐Cheol Yang:** Investigation (equal); project administration (equal); resources (equal). **Amarsanaa Gantsetseg:** Data curation (supporting); formal analysis (equal); visualization (supporting). **Kyeong‐Sik Cheon:** Writing – review and editing (equal). **Ichiro Tamaki:** Methodology (lead); software (equal); visualization (supporting); writing – review and editing (equal). **Jung‐Hyun Lee:** Conceptualization (equal); supervision (lead); visualization (supporting); writing – original draft (supporting); writing – review and editing (equal).

## FUNDING INFORMATION

This research was funded by a research grant from the Baekdudaegan National Arboretum (KIAM‐2021‐KS‐OB‐02‐01‐02).

## CONFLICT OF INTEREST STATEMENT

The authors declare no conflict of interest.

## Supporting information


Figures S1–S2

## Data Availability

The raw dataset analyzed in the current study is available at the GenBank database (BioProject ID PRJNA911453; BioSample accession numbers SAMN32184249–SAMN32184435).
