# Supplementary material for: The genetic structure and demographic history of Zabelia tyaihyonii, endemic to Korean limestone karst forests, based on genome‐wide SNP markers
Source: Ecol Evol. 2023 Jul 3;13(7):e10252. doi: 10.1002/ece3.10252 (PMC10316687; doi:10.1002/ece3.10252)

**Supplementary information**

Figure S1. Mantel test of the relationships among genetic differentiation (*F*_ST_ values) and geographical distance for the 14 populations of *Zabelia tyaihyonii* (*R*^2^ = 0.330, *p* = 0.001).


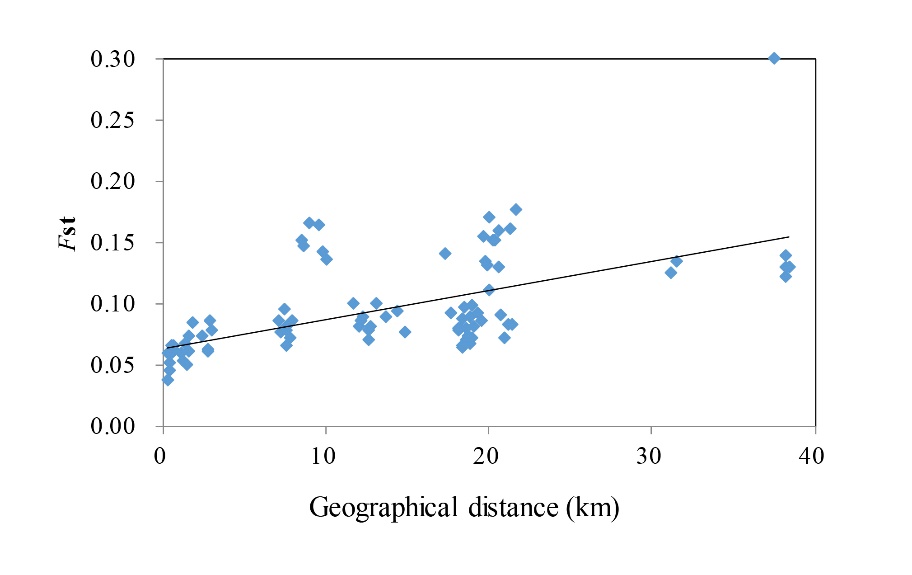


Figure S2. Observed and simulated two-dimensional minor allele site frequency spectra between clusters I and II.


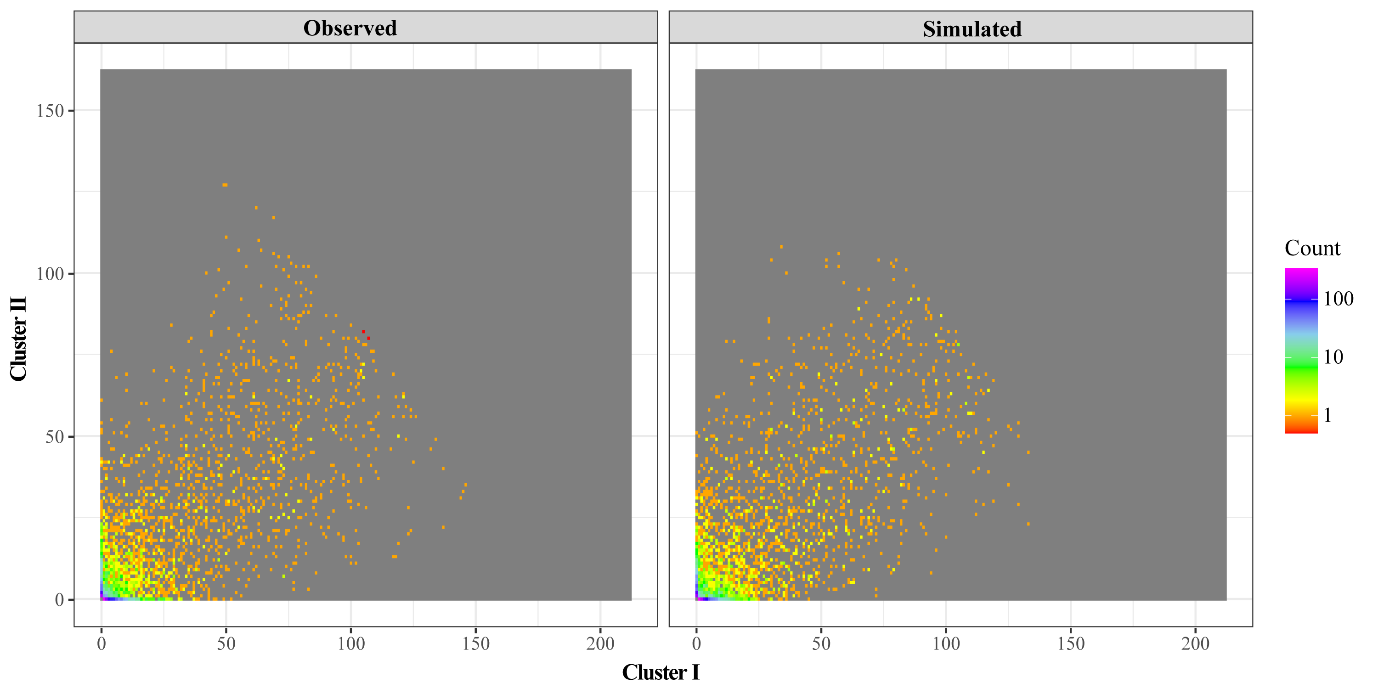

Supplement: Supplementary file 1 — Figures S1–S2 [file ECE3-13-e10252-s001.docx]
